# Effect of lighting illuminance and colour temperature on mental workload in an office setting

**DOI:** 10.1038/s41598-021-94795-0

**Published:** 2021-07-27

**Authors:** Jiayi Bao, Xinbo Song, Yan Li, Yinjie Bai, Qianxiang Zhou

**Affiliations:** 1 School of Biological Science and Medical Engineering, Beihang University, Beijing, 100191 China; 2 Beijing Advanced Innovation Centre for Biomedical Engineering, Beihang University, Beijing, 100191 China; 3 Beijing Aeronautical Technology Research Center, Beijing, 100076 China

**Keywords:** Attention, Psychology

## Abstract

The mental workload of subjects was tested under different lighting conditions, with colour temperatures ranging from 3000 to 6500 K and illuminance ranging from 300 to 1000 lx. We used both psychological and physiological responses for evaluation. The former was based on NASA Task Load Index (NASA-TLX, NASA), and the latter was based on the electroencephalogram (EEG) P3b analysis of event-related potentials using the “oddball” paradigm experimental task. The results show that as illuminance increases, and the response time becomes longer with a colour temperature of 3000 K (P < 0.01). However, when the colour temperature is set at 6500 K, the response time becomes shorter as the illuminance increases (P < 0.01). P3b amplitudes were significantly affected by colour temperature (P = 0.009) and illuminance (P = 0.038) levels. The highest amplitudes occurred at 3000 K and 750 lx, which is consistent with the trend shown by the subjective scale. The data analysis of error rates is not significant. These results suggest that an office environment with a colour temperature of 3000 K and illumination of 750 lx, which exerts the lowest mental workload, is the most suitable for working. However, the interaction between colour temperature and illuminance in affecting the mental workload of participants is not clear. This work provides more appropriate lighting choices with colour temperature and illuminance to reduce people’s mental workload in office settings.

## Introduction

The effects of lighting on health and productivity have raised the level of concern in recent years, as people are spending increasingly more time in artificial light conditions in their everyday lives. Research reports that artificial light has diverse and complex effects on humans, as it affects not only vision but also circadian rhythms^1^. Furthermore, artificial light also impacts migraines^2^, physiological activity levels^3^, mood^4^, etc.

Illuminance and colour temperature are two indicators that can be controlled by artificial lighting, and they also affect human physiology and psychology^5,6^. Illuminance is defined as the luminous flux per square metre, in units of lux (lx). The colour temperature of a lamp equals the thermal temperature that an idealized black body would be required to assume in order to produce the same colour as the lamp, in units of K^7^. Kruithof proposed a diagram that defined comfortable lighting conditions by combining illuminance and correlated colour temperature^8^. He indicated that illuminance and colour temperature have an interactive effect on comfort; thus, through the diagram, we could obtain comfortable illuminance ranges at different colour temperatures. Even though there is not enough evidence to support his study, the idea is regarded as pioneering in the field of lighting characteristics. Based on Kruithof’s diagram, much research has been carried out, which has been focused on adjusting the range of comfortable lighting conditions. Kakitsuba suggested a new diagram of comfortable lighting illuminance and colour temperature based on psychological and physiological responses^9^. The authors used this research method to further analyze the responses of different genders to the comfort of LED lighting^10^. In addition, as a physiological response, electroencephalogram (EEG) is often used to study the effect of light conditions on cognition^11–16^.

Mental workload is the amount of processing capacity that a participant needs to perform a task in a given time^17^. Attention and memory tasks were chosen as the cognitive tasks in the present study, given that attention is one of the most fundamental features of our conscious performance in daily life^18,19^. Among different aspects of attention, sustained attention tasks are closely related to office work and can induce brain electrical activity. Sustained attention involves the ability to maintain concentration on continuous tasks or information sources^20,21^. Thakral et al. and Lee et al. demonstrated that sustained attention influences neural activity in the parietal brain region^22,23^. An uncomfortable working environment can lead to cognitive fatigue and reduced work performance, thereby increasing the mental workload under conditions of sustained attention^24^.

The evaluation method of mental workload includes three aspects: subjective assessment, objective performance, and event-related potential (ERP) analysis. The NASA Task Load Index (NASA-TLX, NASA) is one of the most effective measures of perceived mental workload for subjective assessment. It provides a workload index ranging from 0 to 100 based on six related sources of workload: mental demand, physical demand, temporal demand, performance, effort, and frustration^25^. Thus the subject’s response time and error rate to complete the task are evaluated as objective performance. ERP refers to EEG voltage fluctuations related to physical or mental occurrences over time^26^. The P300 ERP is the maximum positive deflection of the EEG signal that occurs approximately 300 ms after the subject pays attention to the stimulation, including P3a and P3b components^27^. Amplitude (in µv) and latency (in ms) have received widespread attention as evaluations of mental workload^28^. There are many reports that have studied the relationship between P3b component and mental workload through the oddball paradigm^29,30^. It has been reported that the amplitude of P3b decreases with an increase in mental workload^29,31,32^, and its latency increases with an increase in mental workload^30,33^.

In previous studies, the effects of colour temperature on sustained attention have been measured using various tasks, such as word repetition^34^, the Chu Attention Test^35^, short-term memory recall^36^, the Paced Visual Serial Addition Task^37^, and the Numerical Verification Task^38,39^. It has been shown that lighting with warm-white colour temperature usually results in better cognitive performance. Chellappa demonstrated that higher colour temperature is associated with faster reaction times in attention-related tasks^37^. Kocaoğlu found that 4000 K significantly increases the number of errors, while 6500 K is more appropriate for university learning environments^40^. In addition, Min reported that higher illuminance conditions (700 lx) markedly led to lower parietal tonic EEG alpha activity and longer reaction times, than other illuminance conditions (150 lx)^41^. Low-level illuminance often contributes to higher performance appraisals and a better long-term memory rate^42,43^, while high-level illuminance tends to make people less sleepy and more energetic^44^.

However, there is little information in the existing reports about the effect of combinations of illuminance and colour temperature on mental workload. Therefore, we hypothesized that there exists an interaction between illumination and colour temperature that affects mental workload as well as lighting comfort. In addition, the effect of colour temperature and illuminance on mental workload may be nonlinear, but there is a suitable range for relatively low mental workload. The purpose of the present study was to investigate the effects of illuminance and colour temperature on mental workload by EEG and to find appropriate lighting conditions for office environments.

## Results

### Subjective assessments

**Figure 1:**
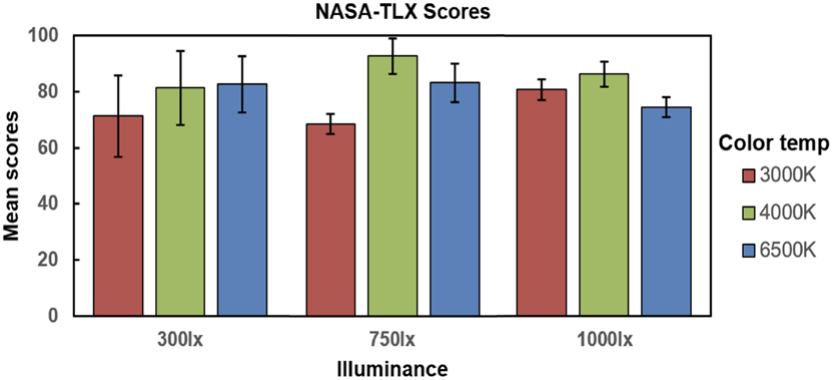
NASA-TLX scores (mean ± SD) under different conditions of colour temperatures (3000 K/4000 K/6500 K) and illuminance (300 lx/750 lx/1000 lx) levels. There was no significant main effect result for either the illuminance or the colour temperature. The interaction effect between illuminance and colour temperatures was also not significant.

The NASA-TLX scores of different lighting conditions are described in Fig. 1. The higher the score is, the greater the mental workload is^45^. The lowest score was obtained at the colour temperature of 3000 K and illuminance of 750 lx. However, there was no significant main effect result for either the illuminance or the colour temperature and the interaction effect between illuminance and colour temperatures was also not significant. A portion of the mean subjective scores are shown in Table 1.

**Table 1:** An example of means ± SD from physiological and psychological response analysis under different lighting conditions.

Colour temperature (K)	Illuminance (lx)	Scores^a^	Re—time^b^	P3b—amp (μv)^c^
3000	300	71.4 ± 14.55	0.12 ± 0.10	0.83 ± 0.30
3000	750	68.6 ± 13.18	0.40 ± 0.22	1.67 ± 0.31
3000	1000	80.9 ± 10.00	0.52 ± 0.24	1.12 ± 0.32
4000	750	92.7 ± 6.36	0.47 ± 0.18	1.25 ± 0.36
6500	750	83.2 ± 4.55	0.65 ± 0.14	0.51 ± 0.18

^a^NASA-TLX scores as psychological response; lower scores predicted lower mental workload.

^b^The min–max–normalized response time; shorter re-time predicted lower mental workload.

^c^P3b amplitude of the P4 electrode; higher P3b – amp predicted lower mental workload.

### Behavioural performance

**Figure 2:**
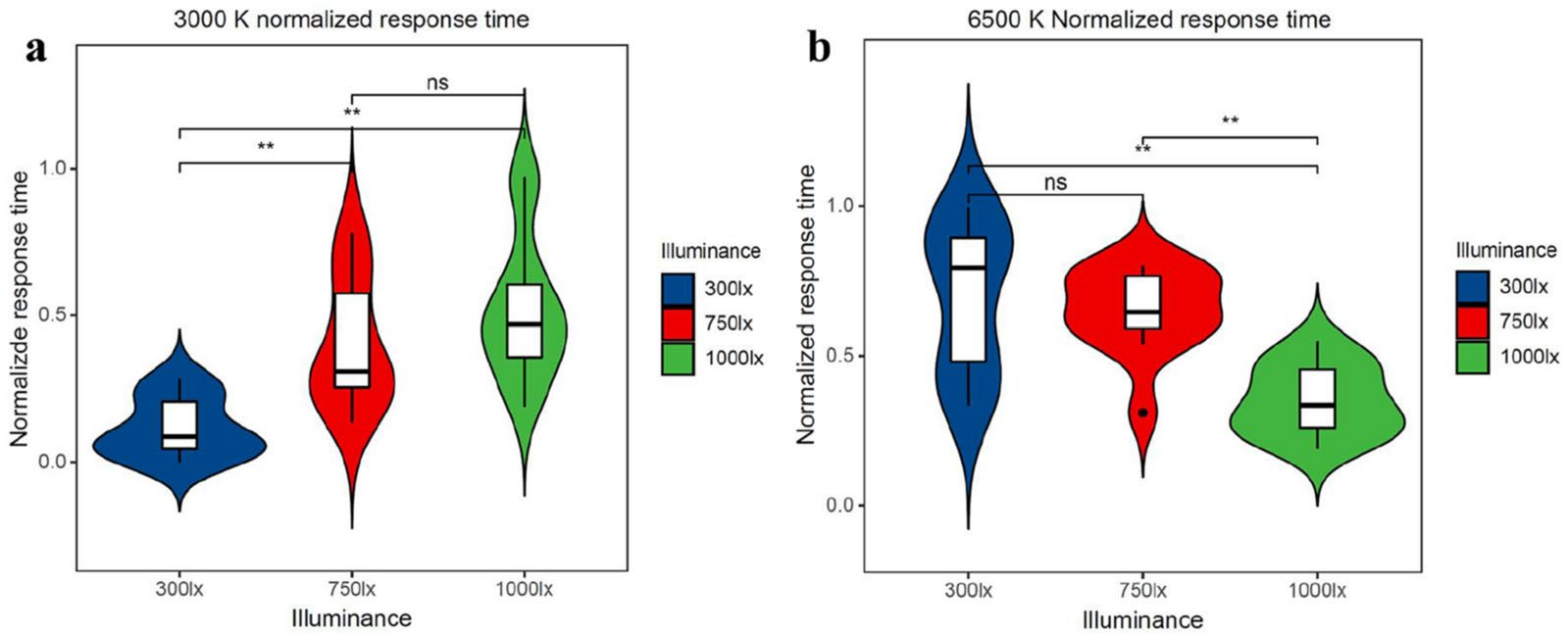
**(a)** The min–max–normalized response time of the experimental task under different illuminances at 3000 K. **(b)** The min–max–normalized response time of the experimental task under different illuminances at 6500 K. The upper diagram shows that the response times of 3000–300 lx and 6500–1000 lx were significantly lower than others (*P < 0.05; **P < 0.01).

The average response time and the accuracy rate in the oddball experimental task were analysed. The accuracy rate was not significantly influenced by illuminance or colour temperature. Moreover, the response time was significantly influenced by the illuminance according to the min–max normalization scores. As shown in Fig. 2a,b, as illuminance increases, the response time becomes longer with a colour temperature of 3000 K (P < 0.01). However, when the colour temperature is set at 6500 K, the response time becomes shorter as illuminance increases (P < 0.01).

### Physiological measurements

The amplitude and latency of P3b with different combinations of colour temperature and illuminance were analysed. Latency was not significantly influenced by illuminance or colour temperature. There was no significant interaction between these factors regarding P3b latency. For P3b amplitude, the analysis of variance on some electrode sites is listed in Table 2. The results indicate that amplitude is significant for the colour temperature (F[2,22] = 5.102, P = 0.009 at CP4) and illuminance levels (F[2,22] = 3.478, P = 0.038 at P4). However, no interaction of colour temperature and illuminance levels was observed (F[4,44] = 2.501, P = 0.053).

**Table 2:** Significance analysis of the influence of colour temperature and illuminance on P3b amplitude.

Source	Dependent variable	F	Sig
Corrected model	C3 amplitude	2.334	.031
	PZ amplitude	2.222	.039
	CP4 amplitude	2.348	.030
Colour temperature × illuminance	C3 amplitude	2.501	.053
Colour temperature	C3 amplitude	3.191	.049
	PZ amplitude	3.970	.025
	CP4 amplitude	5.102	.009
Illuminance	P4 amplitude	3.478	.038

**Figure 3:**
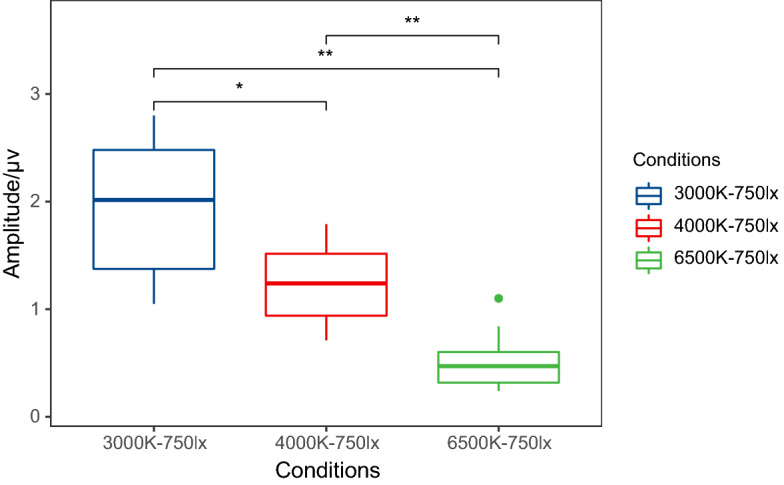
An example of P3b amplitudes with different colour temperatures under 750 lx illuminance at the CP4 electrode (*P < 0.05; **P < 0.01). The amplitude at 3000 K was significantly higher, indicating that the lowest mental workload occurred at 3000 K.

**Figure 4:**
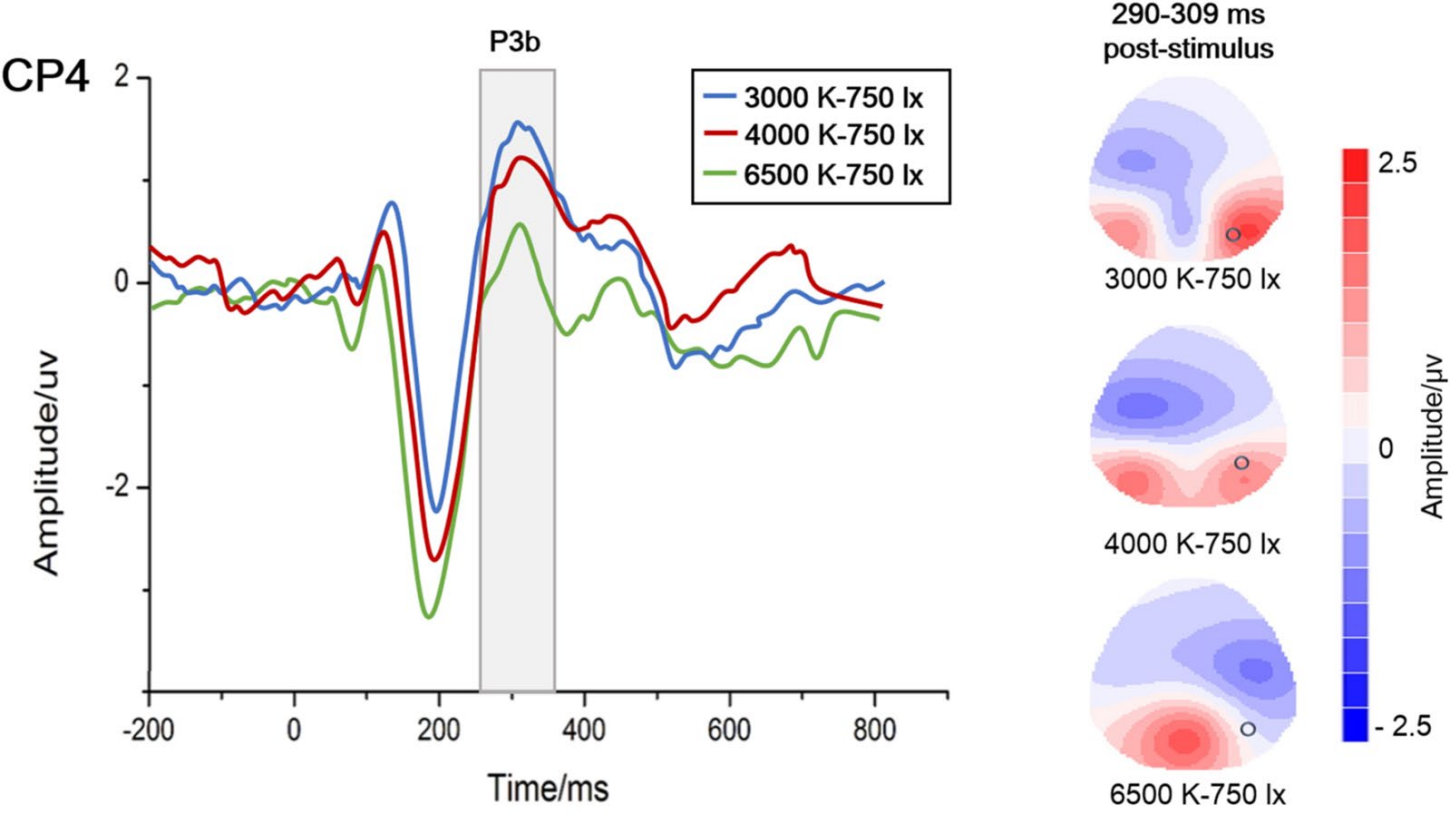
Grand average waveforms of P3b at the CP4 electrode and scalp topographic distributions with different colour temperatures (3000 K/4000 K/6500 K) under 750 lx illuminance. The shaded box shows a significant difference in P3b waves. And the circles at topographic maps represent the CP4 electrode when P3b occurred near 300 ms.

Examples of P3b amplitudes with different colour temperatures under 750 lx illuminance are presented in Fig. 3, and the grand average waveforms of P3b at the CP4 electrode and the scalp topographic distributions are described in Fig. 4. In the case of 750 lx, the results show that the P3b amplitude at CP4 is obviously higher at 3000 K than at 4000 K (P = 0.047) or 6500 K (P = 0.001), meaning that the lowest mental workload occurs at 3000 K.

**Figure 5:**
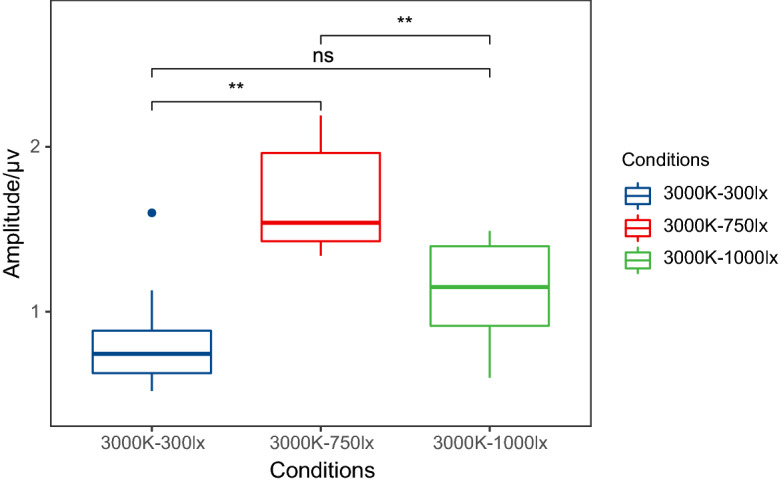
An example of P3b amplitudes with different illuminance levels under 3000 K colour temperature at the P4 electrode (*P < 0.05; **P < 0.01). The amplitude at 750 lx was significantly higher, indicating that the lowest mental workload occurred at 750 lx.

**Figure 6:**
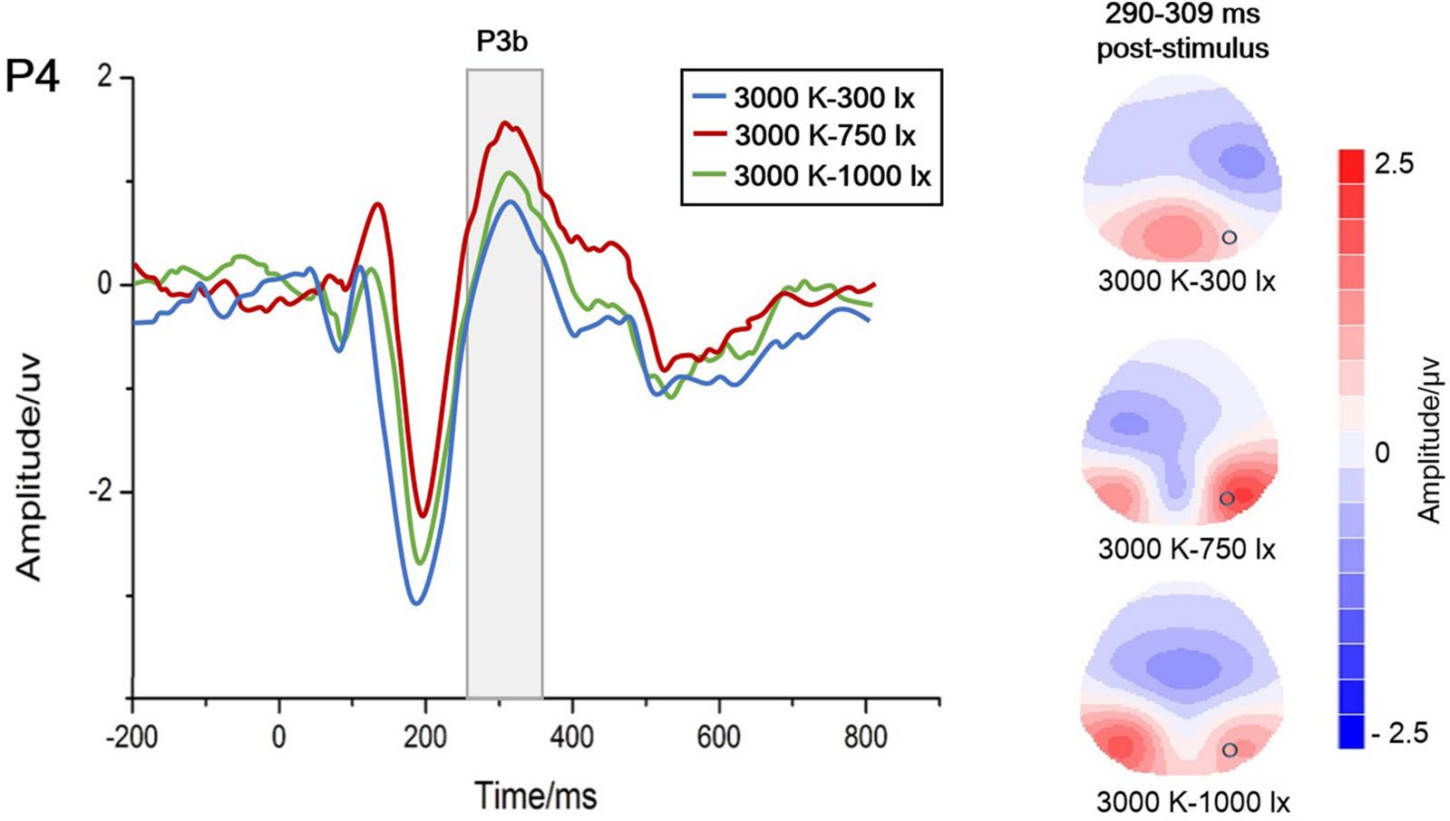
Grand average waveforms of P3b at the P4 electrode and scalp topographic distributions with different illuminance levels (300 lx/750 lx/1000 lx) under 3000 K colour temperature. The shaded box shows a significant difference in P3b waves. And the circles at topographic maps represent the P4 electrode when P3b occurred near 300 ms.

Examples of P3b amplitudes with different illuminance levels under 3000 K colour temperature are displayed in Fig. 5, and the grand average waveforms of P3b at the P4 electrode and the scalp topographic distributions are described in Fig. 6. In the case of 3000 K, the results show that the P3b amplitude at P4 is significantly higher at 750 lx than at 300 lx (P = 0.001) or 1000 lx (P = 0.001), meaning that the lowest mental workload occurs at 750 lx. The means ± SD of P3b amplitudes at 3000 K are listed in Table 1.

## Discussion

The effect of lighting illuminance and colour temperature on mental workload was investigated in the present study by subjective assessment, behavioral performance, and EEG analysis. However, no significant interaction between illuminance and colour temperature on mental workload was found from all aspects of the data, as outlined in Table 2. These results are not consistent with the hypothesis that there would be an interaction between illumination and colour temperature on mental workload, as well as lighting comfort.

Neither illuminance level nor colour temperature shown a significant effect on NASA-TLX scores. The principal reason may be individual differences and the limited sample size.

As indicated in Fig. 2a,b, when the colour temperature is set at 3000 K, the response time will be delayed as the illuminance increases, which is consistent with the findings of a previous study^41^. Yan et al. also found that learning efficiency was higher at a 2700 K colour temperature and 300 lx illuminance^46^. However, the response time becomes shorter as the illuminance increases when the colour temperature is set at 6500 K. We consider 6500 K, which is called a “cool temperature”, to make people more alert and better able to concentrate when the illuminance improves. Chellappa found that light at 6500 K led to significantly faster reaction time in the sustained attention task^37^. Li Lan also reported that greater positive moods were expressed at 2000 lx, 6000 K and 300 lx, 3000 K compared to 2000 lx, 3000 K and 300 lx, 6000 K^47^.

For ERP analysis, a significant effect of light conditions on the P3b latency was not observed. The P3b latency represents the time of mental processing, and it is often increased when the classification of evoked stimuli becomes more difficult^48^. There was no change in the difficulty of the categorizing tasks that elicited stimuli in the present study. The P3b amplitude represents the amount of attention given to evoked stimuli^49^. Different light conditions affect people's ability to focus attention on visual tasks through their impact on the visual system^50^. And the amplitude of P3b decreases with an increase in mental workload^48^. As described in Figs. 3 and 5, P3b amplitudes are observably influenced by colour temperature and illuminance. When the illuminance is 750 lx, the highest amplitude occurs at 3000 K. Manav demonstrated that 2700 K was suggested for ‘relaxation’ and ‘saturation evaluation’^51^. This may indicate that approximately 3000 K is a low mental workload colour temperature. At a colour temperature of 3000 K, the highest amplitude occurs at 750 lx. In either an extremely bright or dark environment, we cannot concentrate and are unable to complete the memory task well. This is the same as the relationship between arousal and attention. The ability to focus attention effectively is nonlinear with arousal. It is roughly like an inverted U-shaped function^52^. Hancock also reported a similar inverted-U law^53^ and that environmental stressors can transform an individual’s adaptability from a stable state to an unstable state. Very low or very high task requirements will reduce adaptability, thereby leading to increased mental workload^54^.

Another purpose of the present study was to find more suitable lighting conditions for the office environment. As described in Table 1, through the analysis of EEG data, a combination of 3000 K colour temperature and 750 lx illumination produced the lowest mental workload. This is also consistent with the results of the subjective assessment.

## Conclusion

Under different lighting conditions of colour temperature (3000 K/4000 K/6500 K) and illuminance (300 lx/750 lx/1000 lx), twelve postgraduates participated in sustained attention task experiments. In addition, NASA-TLX scores as psychological responses, accuracy and response time as behavioral performance, and EEG data as physiological responses were recorded continuously in the experiment. The results show that the combination of 3000 K colour temperature and 750 lx illumination, which requires the lowest mental workload, is better suited to office settings. However, no significant interaction between colour temperature and illumiance for mental workload was found.

This study included only for healthy student participants. Vision problems such as myopia, hyperopia, astigmatism, etc. may lead to deviations in test results. More studies with larger sample sizes are needed to test the findings reported here. In addition, compared with the background lighting conditions, the influence of the screen stimulus light on the experimental results may be more remarkable. The issue of how to set the screen lighting condition to minimize its impact on experimental results needs further research.

## Methods

### Subjects

Twelve postgraduates from Beihang University participated as subjects for this study. All the participants (male, age: 23–27 years, mean 24.3) were right-handed and possessed normal vision. Caffeinated drinks, medication, smoking, and exercise were prohibited before the experiment for one week. Meanwhile, all the participants reported that they slept between six and eight hours the night before the study. The experimental protocol was approved by the Ethics Committee of Beihang University, and all methods were carried out in accordance with relevant guidelines and regulations. All research activities adhered to the principles of the Declaration of Helsinki, and written informed consent was obtained from the participants before the experiment.

### Cognitive task

**Figure 7:**
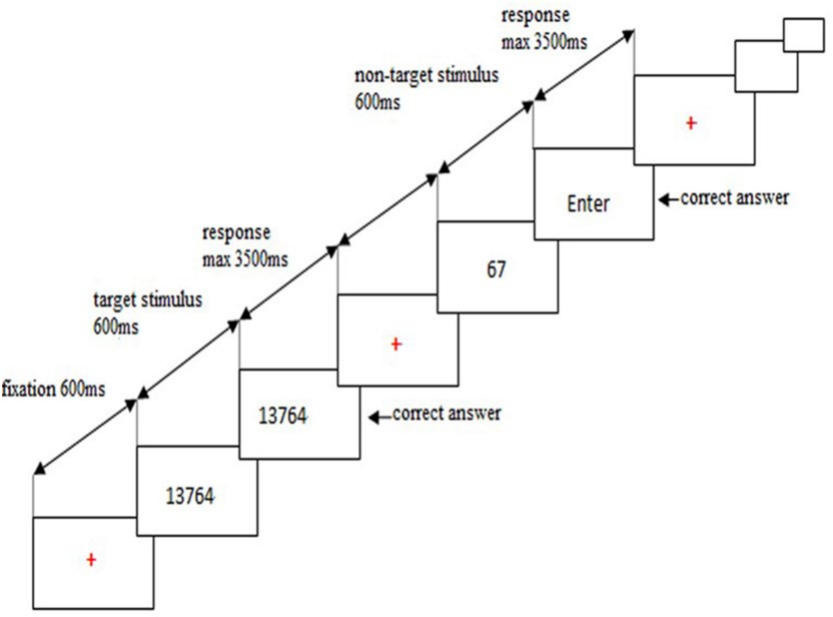
The task flow diagram for a sample stimulus. Stimulus presentation occurred after presentation of a fixation cross.

A computerized task was chosen for this experiment because office workers under artificial lighting obtain the majority of their information from display terminals. The experimental task was a modified “oddball” paradigm controlled by E-prime software (Psychology Software Tools, Inc., Pittsburgh, PA) that placed demands on sustained attention and memory. In this task, the target stimulus was a five-digit number, and the non-target stimulus was a two-digit number. Each block contained 120 target stimuli and 480 non-target stimuli. Between every two target stimuli, there were approximately two to six non-target stimuli. The presentation time for each stimulus was 600 ms, and 3.5 s were allowed for responding stimulus. The task flow diagram is shown in Fig. 7. In the task flow, the start signal occurs in the middle of the computer screen. The ' + ' signal means you should focus, and the sequences of numbers take place in the center of the screen. The image used in the current study was 1280 × 1024 pixels.

Each experimental block corresponding to one illuminance and colour temperature combination took approximately 25 min. Subjects were instructed to memorize each target stimulus number as soon as possible. Then, they were required to enter the number on a form after it disappeared on the computer. If a non-target stimulus appeared, the participants were asked to identify it as such and to press the “Enter” button after it disappeared.

### Procedure

**Figure 8:**
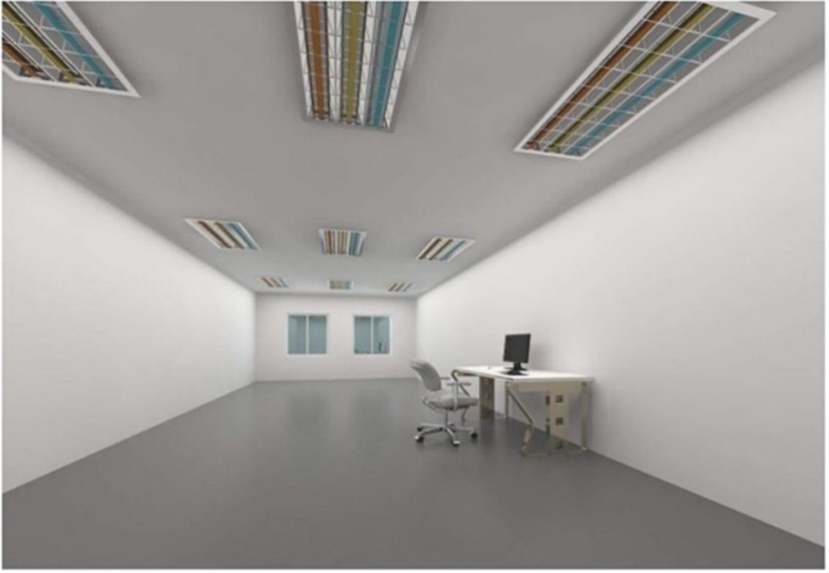
In the laboratory setting, nine controllable luminaires were installed on the ceiling, and black curtains were installed on the windows to block natural sunlight.

The experimental office (dimensions: 10 m L × 4.5 m W × 3.0 m H) is shown in Fig. 8. The air temperature was approximately 25℃. Black curtains were used to exclude natural sunlight. The suspended ceiling was white. The walls were painted light white, and the floor was light grey brick. The lighting system was supplied by nine luminaires that were recessed in the suspended ceiling. The total number of fluorescent lamps (Philips TLD/36 W/830; Philips TLD/36 W/840; Philips TLD/36 W/865) was 27. Each of them was connected to dimmable electronic ballasts, which were regulated by a Light Master 100 lighting control system. The lighting system was designed to provide nine different lighting scenarios, which were numbered on a remote control. This lighting system could be adjusted to maintain constant illumination levels of 300, 750, and 1000 lx on the working surface, while colour temperature of 3000, 4000, and 6500 K could be created separately. Illuminance values were measured by an LMT PO1704 Luxmeter (0—99,999 lx, ± 0.01 lx), and colour temperature values were measured by a TES-136 colourimeter (0–9,999 K, ± 0.02 K). The lighting scenarios in this experiment were in random order to avoid any influence of the sequence. Each experiment consisted of nine trials of 25 min each, followed by breaks of 30 min i.e., enough time for rest and adaptation to the new illumination.

The target stimulus was presented on a PC (Lenovo) screen in front of the participant. In this task, all the participants were required to practice the task until they were skilled at it, or rather when their accuracy rate was 80% or more. The participants were required to focus, to do their best to complete the task as fast as possible, and to ensure the accuracy of their answers. A small reward was given for good performance. During the experiment, the participants’ response times and accuracy rates were recorded by E-prime.

All the participants had 30 min to adapt to the new environment. After that, the participants needed to accomplish the task block. Each subject should complete the NASA-TLX after each block, which was used to evaluate mental workload regarding participants’ subjective feelings. The experiments were conducted from 08:00 to 11:30 and from 14:00 to 18:30. The participants could finish the task in 30 min and rest for 30 min.

### EEG data recording and processing

EEG was recorded by 32 Ag/AgCl electrodes with the international 10/20 system layout mounted in an elastic cap (Easycap, Germany). The signals were recorded by NeuroScan SynAmps amplifiers and Curry 7.0 software (Compumedics NeuroScan, Charlotte, NC), with a 1000 Hz sampling rate. The impedances of all electrodes were kept below 5 kΩ. The reference electrode was FCz, and the ground electrode was placed on the forehead^55^. Additional electrodes were placed at the outer left canthus and below the left eye to measure electrooculography (EOG) activity with a bipolar recording.

EEG data preprocessing was conducted by Curry 7.0 software. The EEG data were first re-referenced to an average of bilateral mastoids, and the bandpass filter was from 0.1 to 30 Hz. Epochs of 1000 ms were obtained for each trial starting 200 ms before the target stimulus onset and ending 800 ms after. A baseline correction was applied (baseline − 200 to 0 ms). A correction algorithm was applied to remove eye movement artefacts (e.g., eye blinks, muscle artefacts) ^56^.

### P3b analysis

In the following we focused on the P3b component elicited by the target stimuli. P3b amplitude and latency were used to infer mental workload under different conditions of colour temperature and illumination. The mean amplitude and latency were calculated at electrode sites (C3, Cz, C4, CP3, CPz, CP4, P3, Pz, and P4).

### Statistics analysis

All variables were first tested for normality with Shapiro–Wilk tests^57^, and all variables followed a normal distribution. Then, the data were analysed with a repeated measures analysis of variance (ANOVA), which included two within-subjects factors (illuminance: 300 lx/750 lx/1000 lx, colour temperature: 3000 K/4000 K/6500 K). The means were compared by Duncan’s test at a probability of 95%. Statistical analyses were performed using SPSS Statistics 17.0 (SPSS, Inc., Chicago, IL, USA). Because of limited numbers of participants, individual differences influenced the raw results. To reduce this influence, the min–max normalization data standardization method that transferred the value of data to a range of [0, 1]^58^ was used to analyse the NASA-TLX and behavioral performance data.

## Data Availability

The data that support the findings of this study are available from the corresponding author upon reasonable request.
